# Supplementary material for: Microbes can help explain the evolution of host altruism
Source: Nat Commun. 2017 Jan 12;8:14040. doi: 10.1038/ncomms14040 (PMC5241693; doi:10.1038/ncomms14040)
Supplement: Supplementary Information — Supplementary figures and Supplementary notes. [file ncomms14040-s1.pdf]

Supplementary Information

Supplementary Note 1 Further analysis of the model

In Supplementary Note 1 we further analyze our model, investigating condition (5) presented in the Methods under several additional parameter regimes, and derive the conditions that allow the fixation of the altruism-inducing microbe. We use the same parameters and model settings as in the main text, unless stated otherwise. Throughout supplementary note 1 we use $\mu = 1 - VT$, $b_m = b$, and $c_m = c$.

In the Methods section we derived condition (5):

$$\text{S1. } p(1-p)\left[T_\alpha - T_\beta + T_\alpha b_g - T_\beta b_g - T_\alpha c_g + T_\beta c_g + (1-\mu)\left(T_\alpha b_m + T_\beta c_m - c_m\right) + \mu\left(T_\alpha b_m p - T_\beta b_m p - T_\alpha c_m p + T_\beta c_m p\right)\right] > 0$$

which determines when altruism-inducing microbes (α) can evolve, and presented results for several special cases. In this section we analyze several additional parameter regimes.

1.1 Case I: α has a lower horizontal transmission probability than β $(T_\alpha < T_\beta)$

If $T_\alpha < T_\beta$, it follows from S1 that p would increase whenever:

$$\text{S2. } p < \frac{T_\alpha - T_\beta - c_m + T_\alpha b_g + T_\alpha b_m - T_\beta b_g - T_\alpha c_g + T_\beta c_g + T_\beta c_m + c_m\mu - T_\alpha b_m\mu - T_\beta c_m\mu}{\mu\left((b_m - c_m)(T_\beta - T_\alpha)\right)}$$

We denote the right hand side of S2 by R and note that when (i) $R \leq 0$, S2 cannot be satisfied since p is non-negative, and thus p does not increase, meaning that altruism-inducing microbes do not evolve; (ii) if $R \geq 1$, S2 is always satisfied since $p \leq 1$, and thus p increases, meaning that altruism-inducing microbes will fixate in the population; and (iii) if $0 < R < 1$, S2 will be

satisfied for only some values of p, meaning that altruism-inducing microbes will neither fixate nor go to extinction. In this case we reach polymorphism, which is stable, as we show below.

We first consider the regime of $\mu \geq \frac{T_\alpha}{T_\beta}$.

(i) Microbe-induced altruism will go to extinction when $R \leq 0$. This happens when:

$$\text{S3.}\ \frac{b_m}{c_m} \leq \frac{(T_\beta - T_\alpha)(1 + b_g - c_g)}{c_m T_\alpha (1-\mu)} + \frac{1 - T_\beta}{T_\alpha}$$

(ii) Microbe-induced altruism reaches fixation when $R \geq 1$. However, for this regime of μ, R is never greater than 1. To see why, note that $R \geq 1$ if and only if:

$$\text{S4.}\ \frac{b_m}{c_m} \leq \frac{(T_\beta - T_\alpha)(1 + b_g - c_g) + c_m(1 - T_\beta) - c_m \mu (1 - T_\alpha)}{c_m (T_\alpha - \mu T_\beta)}$$

Since $\mu \leq 1$ and $c_g + c_m < 1$, the numerator of the right hand side of S4 is positive:

$$(T_\beta - T_\alpha)(1 + b_g - c_g) + c_m(1 - T_\beta) - c_m\mu(1 - T_\alpha) > (T_\beta - T_\alpha)(1 + b_g - c_g) + c_m(1 - T_\beta) - c_m(1 - T_\alpha)$$
$$= (T_\beta - T_\alpha)(1 + b_g - c_g - c_m) > 0$$

Since also $\mu > \frac{T_\alpha}{T_\beta}$, the denominator of the right hand side of S4 is negative. Therefore, S4 cannot be satisfied and hence R is not greater than 1.

(iii) Polymorphism therefore exists when:

$$\text{S5.}\ \frac{b_m}{c_m} > \frac{(T_\beta - T_\alpha)(1 + b_g - c_g)}{c_m T_\alpha (1-\mu)} + \frac{1 - T_\beta}{T_\alpha}$$

Next, we consider the case of $\mu < \frac{T_\alpha}{T_\beta}$.

(i) The condition under which microbe-induced altruism will go extinct ($R \leq 0$), is S3 as above.

(ii) Microbe-induced altruism fixates ($R \geq 1$), independent of its starting condition, when:

$$S6.\ \frac{b_m}{c_m} \geq \frac{(T_\beta - T_\alpha)(1+b_g-c_g)+c_m(1-T_\beta)-c_m\mu(1-T_\alpha)}{c_m(T_\alpha-\mu T_\beta)}$$

(iii) Combining conditions S3 and S6, we find that polymorphism exists when:

$$S7.\ \frac{(T_\beta - T_\alpha)(1+b_g-c_g)}{c_m T_\alpha(1-\mu)} + \frac{1-T_\beta}{T_\alpha} < \frac{b_m}{c_m} < \frac{(T_\beta - T_\alpha)(1+b_g-c_g)+c_m(1-T_\beta)-c_m\mu(1-T_\alpha)}{c_m(T_\alpha-\mu T_\beta)}$$

1.2 Case II: $\boldsymbol{\beta}$ has a lower horizontal transmission probability $(\boldsymbol{T_\alpha > T_\beta})$

When $T_\alpha > T_\beta$, $\mu < \frac{T_\alpha}{T_\beta}$ is always satisfied since $\mu \leq 1$.

In analogy to section (1.1), the conditions for the spread of altruism in the case $T_\alpha > T_\beta$ are:

α will go extinct for any $0 < p < 1$, when:

$$S8.\ \frac{b_m}{c_m} \leq \frac{c_m(1-T_\beta)-c_m\mu(1-T_\alpha)-(T_\alpha - T_\beta)(1+b_g-c_g)}{c_m(T_\alpha-\mu T_\beta)}$$

α will spread in the population for any $0 < p < 1$, i.e. will fixate, when:

$$S9.\ \frac{b_m}{c_m} \geq \frac{1-T_\beta}{T_\alpha} - \frac{(T_\alpha - T_\beta)(1+b_g-c_g)}{c_m T_\alpha(1-\mu)} \quad \text{(for } \mu < 1\text{; and for any } b_m/c_m \text{ when } \mu = 1\text{)}$$

Combining S8 and S9, polymorphism is expected when:

$$\frac{c_m(1-T_\beta)-c_m\mu(1-T_\alpha)-(T_\alpha - T_\beta)(1+b_g-c_g)}{c_m(T_\alpha-\mu T_\beta)} < \frac{b_m}{c_m} < \frac{1-T_\beta}{T_\alpha} - \frac{(T_\alpha - T_\beta)(1+b_g-c_g)}{c_m T_\alpha(1-\mu)}$$

We note that when $T_\alpha > T_\beta$, α can in some cases spread in the population due to its infectivity, even for $b_m < c_m$. However, we do not focus on this case since it's not in the scope of the prisoners' dilemma setting.

1.3 Polymorphism analysis

We showed above that when the horizontal transmission rates are unequal, polymorphism exists for some parameter regimes. In this sub-section, we further analyze these polymorphisms.

We first investigate the polymorphism in the case $T_\alpha < T_\beta$. As shown above, polymorphism exists when condition S5 or S7 are satisfied (depending on the regime of μ). In order to examine if it is a stable polymorphism we can differentiate the left side of S1 with respect to p and calculate the derivative at steady state. We denote the left hand side of inequality S1 by f. It can be seen that $f(p) = p(1-p) \cdot g(p)$, for a $g(p)$ that is linear in p. Therefore, f is a cubic function of p, and thus can have only three real roots. It is easy to see that 0 and 1 are roots of f, hence there can be only one polymorphic root in the range $(0,1)$.

We begin by differentiating g:

$$\text{S10. } g'(p) = -\mu\left(T_\beta - T_\alpha\right)(b_m - c_m)$$

Therefore, when differentiating f we get:

$$\text{S11. } f'(p) = (1-2p) \cdot g(p) - p(1-p)\mu\left(T_\beta - T_\alpha\right)(b_m - c_m)$$

Polymorphism exists if there is $0 < p^* < 1$ such that $g(p^*) = 0$. For such p^* we get:

$$\text{S12. } f'(p^*) = -p^*(1-p^*)\mu\left(T_\beta - T_\alpha\right)(b_m - c_m)$$

When $T_\alpha < T_\beta$, $f'(p^*)$ for such p^* is always negative. In addition, since $0 < p^*, \mu, T_\alpha, T_\beta, b_m, c_m < 1$, we get that $0 < p^*(1-p^*) < \frac{1}{4}$ and $0 < (b_m - c_m), \left(T_\beta - T_\alpha\right) < 1$ and therefore we can conclude that $f'(p^*) > -\frac{1}{4}$. Thus we see that when $T_\alpha < T_\beta$ the polymorphic equilibrium is stable whenever it exists.

Using an analogous analysis, we find that for $T_\alpha > T_\beta$ the polymorphism is unstable, and therefore any perturbation from the steady state will lead to fixation or extinction.

Supplementary Figure 1 examines the effect of the various parameters on the range of polymorphism.

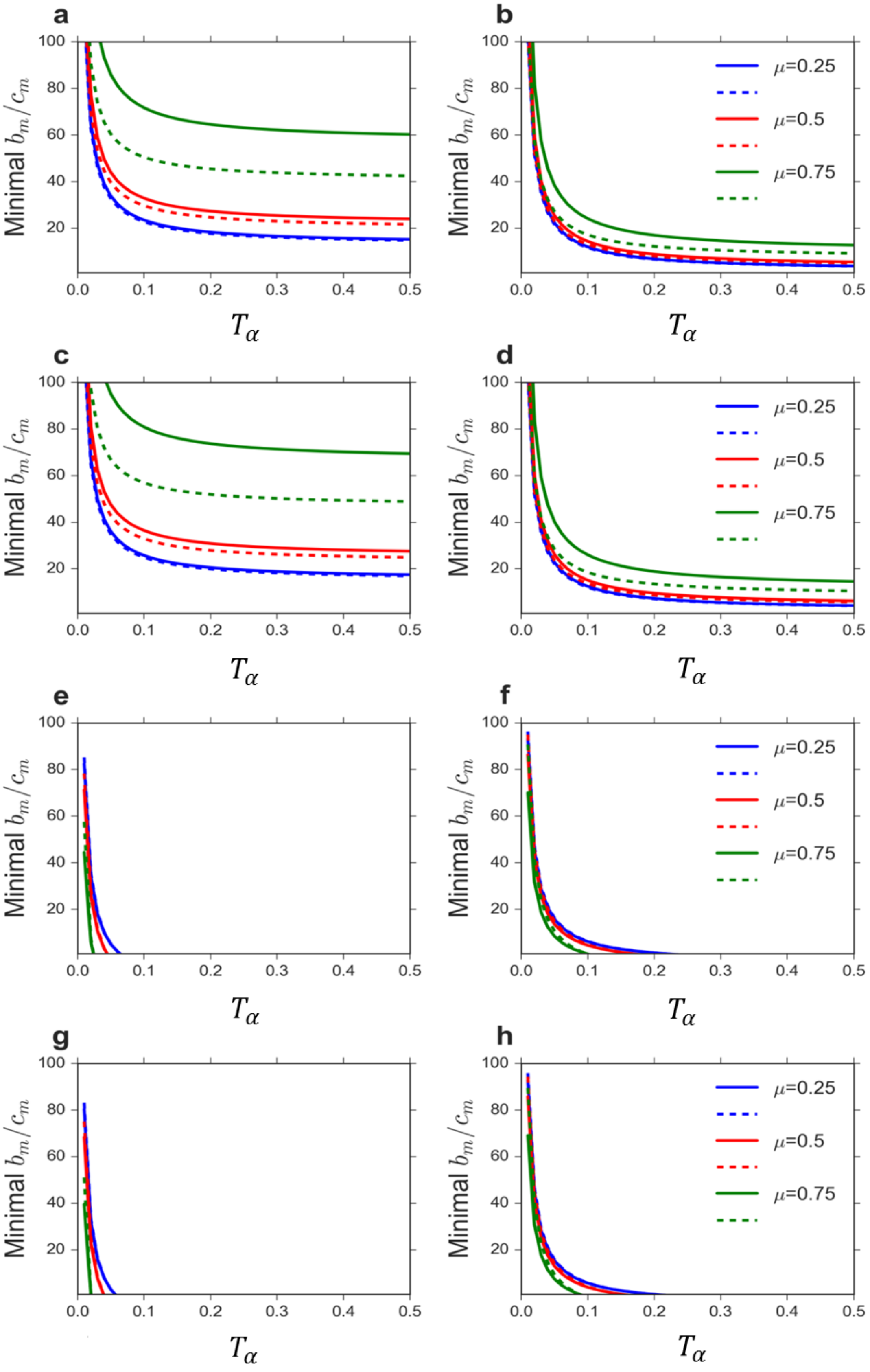

a
b
c
d
e
f
g
h
Minimal b_m/c_m
T_α
μ=0.25
μ=0.5
μ=0.75

Supplementary Figure 1: The effect of μ on the b_m/c_m thresholds and polymorphism range. We plot the upper threshold (solid line; for b_m/c_m above this line altruism fixates) and the lower threshold (dashed line; for b_m/c_m beneath this line altruism goes extinct) for the case of $T_\beta = 1.1T_\alpha$ (figures **a, b, c, d**) and $T_\beta = 0.9T_\alpha$ (figures **e, f, g, h**). We examined several parameters of cost and benefit: $c_g = 0.01, b_g = 0.05$ (figures **a, b, e, f**), $c_g = 0.05, b_g = 0.25$ (figures **c, d, g, h**), $c_m = 0.01$ (figures **a, c, e, g**) and $c_m = 0.05$ (figures **b, d, f, h**). It can be seen that the gap between the thresholds (which is where polymorphism exists) becomes significant only for high μ and low c_m. It is also shown that when $T_\alpha < T_\beta$ (figures **a, b, c, d**), b_m/c_m needed for fixation of α increases with μ, but when $T_\alpha > T_\beta$ (figures **e, f, g, h**), b_m/c_m needed for fixation of α decreases with μ.

1.4 The effect of genetic background of altruistic behavior among the hosts

We consider the effect of host genes for altruistic behavior ($c_g, b_g > 0$) in the case of perfect vertical transmission ($\mu = 0$). Inequality (8), derived in the Methods, describes the condition for the spread of microbe-induced altruism in the presence of genetic background for altruism:

$$\text{S13. } T_\alpha b_m > c_m(1 - T_\beta) + (1 + b_g - c_g)(T_\beta - T_\alpha)$$

Based on S13, Supplementary Figure 2 shows the effect of a fixed genetic background of altruism among the hosts on the fixation of the altruism-inducing microbes.

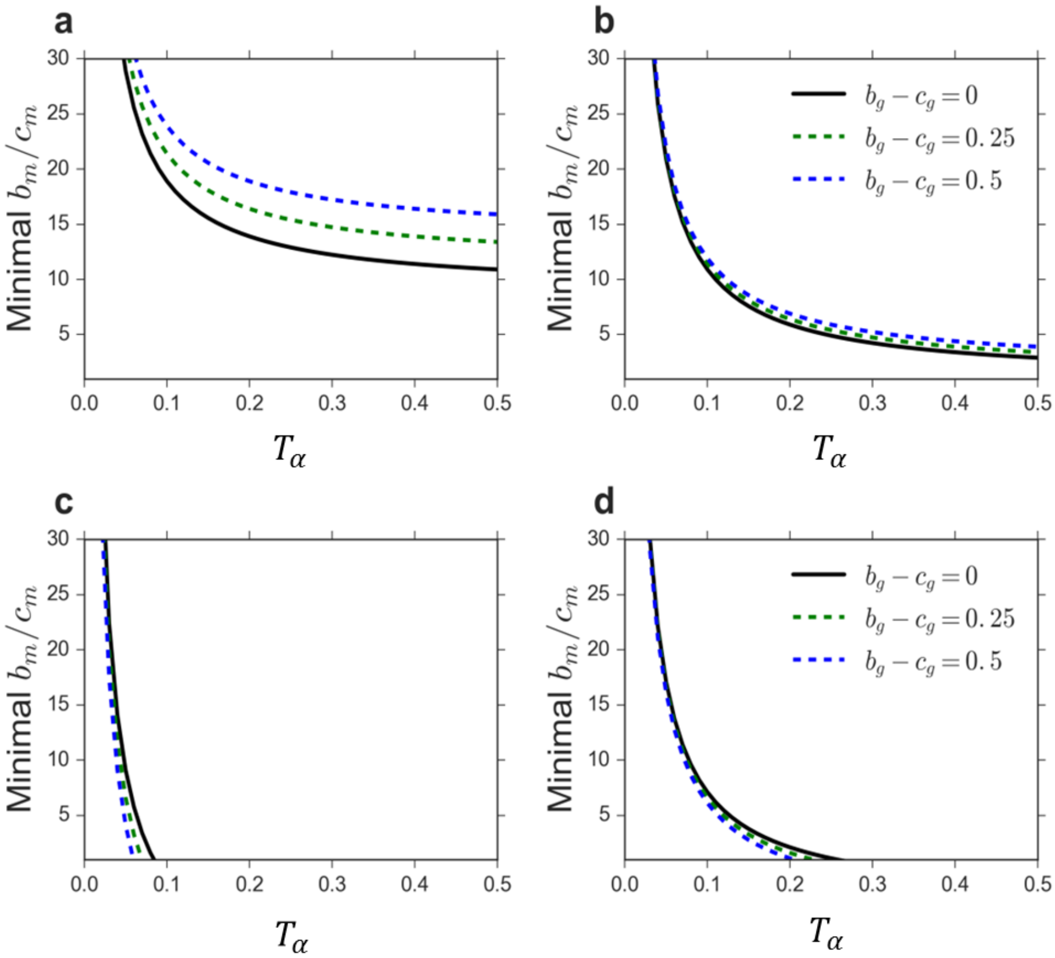

Supplementary Figure 2: The effect of genetic altruistic background on the b_m/c_m threshold needed for fixation of α. We plot the b_m/c_m threshold above which altruism spreads for various $b_g - c_g$ values, in the case of perfect vertical transmission ($\mu = 0$). We show results for $T_\beta = 1.1T_\alpha$ (figures **a, b**), $T_\beta = 0.9T_\alpha$ (figures **c, d**), $c_m = 0.01$ (figures **a, c**) and $c_m = 0.05$ (figures **b, d**).

1.5 Rate of α's spread as a function of vertical transmission

In this section we analyze the rate of change in the proportion of microbe α in the population, for the case of no genetic background of altruistic behavior in the population ($b_g = 0, c_g = 0$) and equal horizontal transmission probability ($T_\alpha = T_\beta = T$). Under these assumptions, equality (4) from the Methods section becomes:

$$S14.\ p' = \frac{1}{\overline{\omega}}[p^2(1+b_m-c_m)(1-\mu q) + pq(1-T)(1-c_m)(1-\mu q)$$
$$+pqT(1+b_m)(1-\mu q) + pqT(1-c_m)\mu p + pq(1-T)(1+b_m)\mu p + q^2\mu p]$$

and $\overline{\omega} = 1 + p(b_m - c_m)$. We define $\Delta p = p' - p$ and we derive Δp with respect to μ.

$$S15.\ \frac{\partial \Delta p}{\partial \mu} = \frac{1}{\overline{\omega}}(-p^2q(1+b_m-c_m) - pq^2(1-T)(1-c_m) - pq^2T(1+b_m) + p^2qT(1-c_m) + p^2q(1-T)(1+b_m) + q^2p)$$

When $\frac{\partial \Delta p}{\partial \mu} < 0$, the rate of change in the frequency of hosts carrying α decreases as μ increases. After simplifying S15 we find that $\frac{\partial \Delta p}{\partial \mu} < 0$ when:

$$S16.\ p(p-1)(Tb_m - c_m + Tc_m) < 0$$

and for $0 < p < 1$ we get:

$$S17.\ \frac{b_m}{c_m} > \frac{1-T}{T}$$

equivalent to condition (1) in the main text when horizontal transmission is equal. That is, whenever the altruism-inducing microbe (α) can evolve (condition (1)), it follows from S17 that its rate of spread increases with the probability of vertical transmission, $VT = 1 - \mu$.

Supplementary Note 2 Model analysis based on the fitness of the two microbes

In the Methods section we derive the condition for fixation of altruism-inducing microbes in a population by analyzing the recursion equation describing the proportion of altruists in the next generation. In this section we show a different approach for the derivation, through analysis of

the inclusive fitness of both microbes. Since microbes are transferred vertically, their fitness is affected by their host fitness. In addition, the microbes' fitness is affected by their horizontal transmission probability. We focus on the special case of perfect vertical transmission $(VT = 1)$ and no genetic background of altruistic behavior $\left(b_g = c_g = 0\right)$, and denote $b_m = b$, and $c_m = c$. We derive the same condition (1) presented in the main text.

In order to consider the fitness of the altruism-inducing microbes, we consider all possible interactions involving a host carrying α (termed "altruist" below). We assume that the microbes are present only within hosts, and the fitness of a microbe is the expected number of hosts in the next generation, who are infected by the offspring of this microbe. The baseline fitness is set to 1 for both microbes:

First, the altruist may interact with another altruist, with probability p, and in such an interaction the fitness of each altruist is: $1 + b_m - \mathrm{c_m}$.

Second, the altruist may interact with a "selfish individual" (host carrying β), with probability q. Then, the probability of no horizontal transmission during interaction is $(1 - T_\alpha)\left(1 - T_\beta\right)$, and in that case the fitness of the altruist is: $1 - c_m$; The probability that only the altruist infects its partner is $T_\alpha\left(1 - T_\beta\right)$ and in this case we now have two altruists with fitnesses $1 + b_m$ and $1 - c_m$; The probability that only the "selfish individual" infects its partner is $T_\beta(1 - T_\alpha)$, and in this case we now have two "selfish individuals"; Finally, the probability that both individuals infect each other is $T_\beta T_\alpha$, and in this case we now have an altruist with fitness: $1 + b_m$.

Thus, the fitness of an altruism inducing microbe (α) is:

$$\text{S18. } \omega_\alpha = p(1 + b_m - c_m) + q\left((1 - T_\alpha)\left(1 - T_\beta\right)(1 - c_m) + T_\alpha\left(1 - T_\beta\right)(2 + b_m - c_m) + T_\alpha T_\beta(1 + b_m)\right)$$

Similarly, we can calculate the fitness of microbe β:

$$S19.\ \omega_\beta = q + p\left((1-T_\alpha)(1-T_\beta)(1+b_m) + T_\beta(1-T_\alpha)(2+b_m-c_m) + T_\alpha T_\beta(1-c_m)\right)$$

Altruism spreads when $\omega_\alpha > \omega_\beta$. That is, when:

$$T_\alpha b_m > c_m(1-T_\beta) + (T_\beta - T_\alpha)$$

This is the same condition that was derived in the Methods (9) and presented in the Results as condition (1).

Supplementary Note 3 Microbe-induced altruism in the presence of host polymorphism for altruism

Up until now we assumed that the host population is homogenous in the locus that determines altruistic behavior. In Supplementary Note 3 we consider a population that is polymorphic with respect to both altruism-inducing host genes and altruism-inducing microbes. We show that altruism encoded in the host genes does not evolve in this model irrespective of the presence of microbe α, while microbe-induced altruism can evolve, irrespective of the presence of altruism encoded in the host's genes. The results are therefore identical to the ones obtained for microbe-induced altruism alone.

Individuals with allele A act altruistically: when interacting with another individual they pay a fitness cost c_g, while the receiver gets a fitness benefit b_g. Individuals with allele E do not behave altruistically.

In addition, each individual in the population carries one of two microbe types. Microbes of type α manipulate their host to act altruistically: A host carrying α pays a fitness cost c_m during interaction, while its partner gets a fitness benefit b_m. Microbes of type β do not affect their host's behavior.

We assume independent and additive effects of the microbes and the host's genetics on the host behavior. For example, if an individual has an altruistic allele, and it also carries the

altruism-inducing microbe (type $A\alpha$), it pays a fitness cost of $c_g + c_m$ during interactions, while its partner receives a fitness benefit of $b_g + b_m$.

In this setup we now have four types of individuals:

$A\alpha$ – carry both an allele for altruism (b_g, c_g) and altruism-inducing microbes (b_m, c_m).

$E\alpha$ – do not carry an allele for altruism, but carry altruism-inducing microbes (b_m, c_m).

$A\beta$ – carry an allele for altruism (b_g, c_g), but not altruism-inducing microbes.

$E\beta$ – carry neither an allele for altruism, nor altruism-inducing microbe.

Payoff matrix:

	$\boldsymbol{A\alpha}$	$\boldsymbol{E\alpha}$	$\boldsymbol{A\beta}$	$\boldsymbol{E\beta}$
$\boldsymbol{A\alpha}$	$b_g - c_g + b_m - c_m$	$-c_g + b_m - c_m$	$b_g - c_g - c_m$	$-c_g - c_m$
$\boldsymbol{E\alpha}$	$b_g + b_m - c_m$	$b_m - c_m$	$b_g - c_m$	$-c_m$
$\boldsymbol{A\beta}$	$b_g - c_g + b_m$	$-c_g + b_m$	$b_g - c_g$	$-c_g$
$\boldsymbol{E\beta}$	$b_g + b_m$	b_m	b_g	0

We denote by p_A, p_E, q_A, q_E the proportions of $A\alpha, E\alpha, A\beta, E\beta$ in the population, respectively, and calculate the mean fitness of the population. Similar to the derivation of the mean fitness in the Methods (eq. (3)), we derive the mean fitness in this generalized case:

$$\text{S20. } \bar{\omega} = 1 + p_A(b_g + b_m - c_g - c_m) + p_E(b_m - c_m) + q_A(b_g - c_g)$$

Now we can derive the proportion of each type in the next generation:

$$\text{S21. } p_A{}' = \frac{1}{\bar{\omega}} \cdot \left(p_A^2(1 + b_g - c_g + b_m - c_m) + p_A p_E(1 - c_g + b_m - c_m) + p_A q_A(1 - T_\beta)(1 + b_g - c_g - c_m) + p_A q_E(1 - T_\beta)(1 - c_g - c_m) + p_A q_A T_\alpha(1 + b_g - c_g + b_m) + p_E q_A T_\alpha(1 - c_g + b_m)\right)$$

$$S22.\ p_E{}' = \frac{1}{\bar{\omega}} \cdot \Big(p_E p_A (1 + b_g + b_m - c_m) + p_E^2 (1 + b_m - c_m) + p_E q_A (1 - T_\beta)(1 + b_g - c_m) + p_E q_E (1 - T_\beta)(1 - c_m) + p_E q_E T_\alpha (1 + b_m) + p_A q_E T_\alpha (1 + b_g + b_m) \Big)$$

$$S23.\ q_A{}' = \frac{1}{\bar{\omega}} \cdot \Big(q_A p_A (1 - T_\alpha)(1 + b_g - c_g + b_m) + q_A p_E (1 - T_\alpha)(1 - c_g + b_m) + q_A^2 (1 + b_g - c_g) + q_A q_E (1 - c_g) + q_A p_A T_\beta (1 + b_g - c_g - c_m) + q_E p_A T_\beta (1 - c_g - c_m) \Big)$$

$$S24.\ q_E{}' = 1 - p_A{}' - p_E{}' - q_A{}'$$

Numerical analysis of these equations shows that regardless of the initial proportions in the population and the b_g, c_g values, the only types that fixate in the population are $E\alpha$ and $E\beta$, namely, the host allele for altruism always goes extinct. The results are identical to the results of the main model without the host alleles A and E: $E\alpha$ goes to fixation in the exact same parameters that α alone goes to fixation according to inequality (1), presented in the main text (Fig. 2, compare with Supplementary Figure 3).

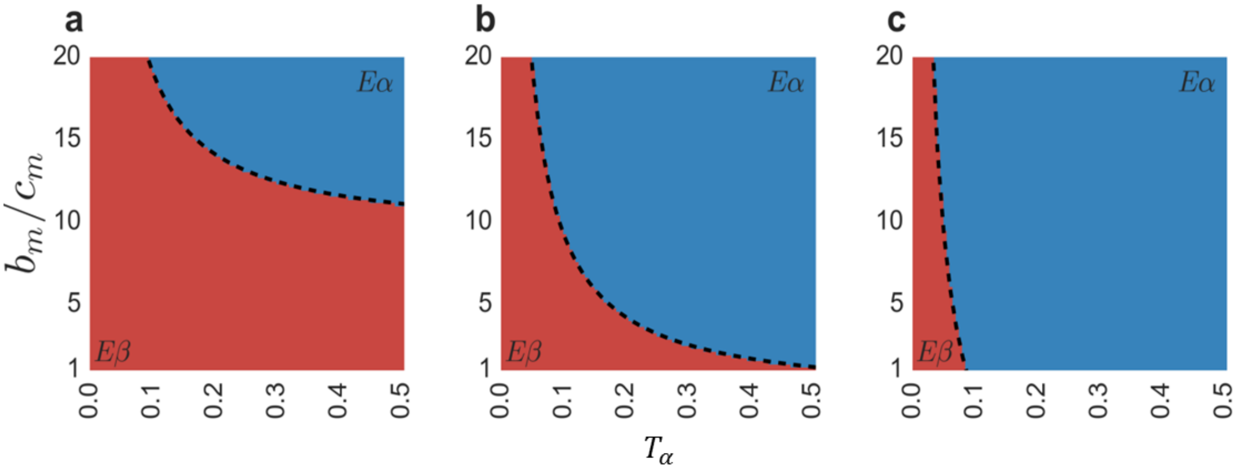

Supplementary Figure 3: Fixation of the microbes is independent of the host alleles. A phase diagram of the type that reaches fixation is plotted as a function of α's horizontal transmission probability and b_m/c_m ratio, for $c_m = 0.01$ and several horizontal transmission ratios: $T_\beta = 1.1T_\alpha$ **(a)**, $T_\beta = T_\alpha$ **(b)** and $T_\beta = 0.9T_\alpha$ **(c)**. Blue areas represent parameter regimes where $E\alpha$ fixates, while red areas represent parameters where $E\beta$ fixates. The results are based on numerical analysis of equations S20-S24. The dashed lines show the critical value derived from condition (1) in the main text (also presented in Fig. 2a). The threshold for the fixation of α, derived from the

numerical analysis, is identical to inequality (9) in the methods. The same results were obtained for a wide range of initial proportions of the different individual types, and various b_g, c_g values.

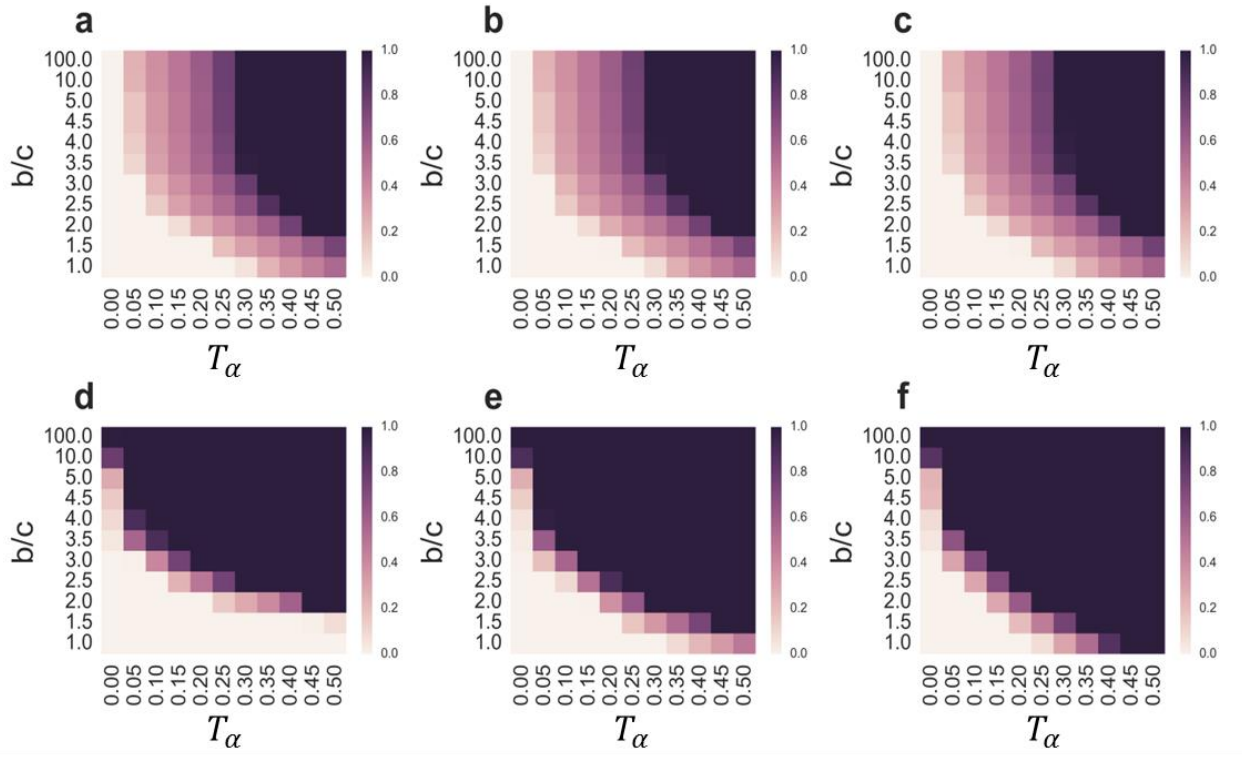

Supplementary Figure 4: Microbe-induced altruism can flourish in a spatial Prisoners' Dilemma scenario, even when it has horizontal transmission disadvantage. Hosts carrying either microbes of type α or β are placed on a 100×100 lattice grid. Hosts carrying microbe α initially inhabit 5% of the sites, chosen in random positions in the lattice. The final proportion of hosts that carry microbe α is plotted (color coded) as a function of horizontal transmission probability $T_\alpha = T_\beta = T$ and b/c values, for $K = 1$ (**a, b, c**), $K = 8$ (**d, e, f**), $T_\beta = 1.1T_\alpha$ (**a**, **d**), $T_\beta = T_\alpha$ (**b**, **e**) and $T_\beta = 0.9T_\alpha$ (**c**, **f**). Each cell in the plots represents the mean of at least 100 runs. For $K = 1$ (a single interaction per individual per generation) we find that a mild shift from $T_\alpha = T_\beta$ into the cases of $T_\beta = 0.9 \cdot T_\alpha$ (horizontal transmission advantage to α) and $T_\beta = 1.1 \cdot T_\alpha$ (horizontal transmission advantage to β) has a very minor effect on the results. When we increase the number of interactions to $K = 8$, the same change in horizontal transmission ratio has a somewhat larger effect, as the same rate of horizontal transmission is applied 8 times in each generation. As can be seen, even when α has a horizontal transmission disadvantage, it can still reach stable polymorphism or fixation (figures **a**, **d**).

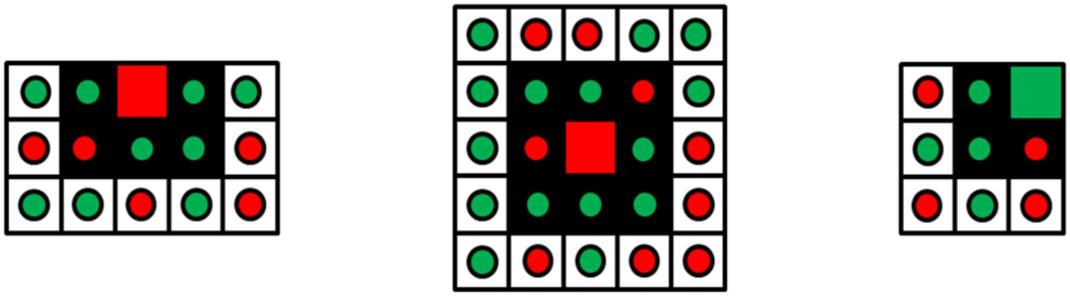

Supplementary Figure 5: Individuals can interact only with their immediate neighbors. Example of interactions: the focal individual (square) can interact only with its immediate neighbors (black background). There are usually eight neighbors, unless the focal individual is close to one of the grid edges.

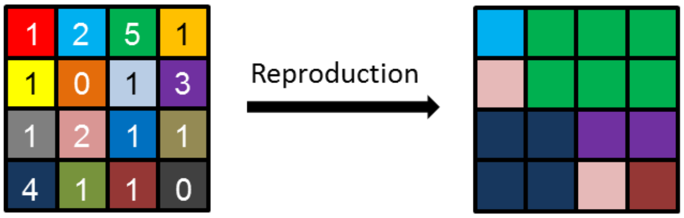

Supplementary Figure 6: Example of the reproduction procedure in the simulations. Reproduction was modeled after Nowak and May (1992). A new lattice grid of the same size is formed. Every site in the new lattice is inhabited by a replicate of the fittest host from the same location, and its immediate neighborhood, in the original lattice. If there are multiple hosts with the same maximal fitness in the neighborhood, the parent is chosen at random from the fittest hosts.
